# 

**Published:** 1850-12

**Authors:** 


### CONTENTS

OF THE

### MEDICAL EXAMINER.

NEW SERIES—NO. LXXII.—DECEMBER, 1850.

### ORIGINAL COMMUNICATIONS.

Fragments from the note-book of an office pupil of the late Dr. George M'Clellan, containing cases and clinical remarks, - - 687
Remarks on the Medical Topography of Callao and Lima. By G. R. B. Horner, M. D., Fleet Surgeon, Pacific Squadron, - - 694
Case of Idiopathic Tetanus. By D. G. Gregory, M. D., of La Grange, Texas, - - - - - - - - 702
Pennsylvania Hospital—Surgical Wards—Service of Dr. Norris. Cases discharged from Jan. 1st to Feb. 1st, 1850, - - 705
Pennsylvania Hospital—Surgical Wards—Service of Dr. Norris. Cases discharged from Feb. 1st to March 1st, 1850, - - 706
Pennsylvania Hospital—Surgical Wards—Service of Dr. Peace. Cases discharged from March 1st to April 1st, 1850, - - 707
Pennsylvania Hospital—Surgical Wards—Service of Dr. Peace. Cases discharged from April 1st to May 1st, 1850, - - 708

### BIBLIOGRAPHICAL NOTICES.

Transactions of the American Medical Association, instituted 1847, - 709
The Races of Men—A Fragment. By Robert Knox, M. D., Lecturer on Anatomy, and Corresponding Member of the National Academy of Medicine of France, - - - - 716
Of the causes, nature and treatment of Palsy and Apoplexy: of the forms, seats, complications and morbid relations of paralytic and apoplectic diseases. By James Copland, M. D., F. R. S., &c. &c., - - - - - - - 723
Household Surgery, or Hints on Emergencies. By John F. South, one of the Surgeons to St. Thomas' Hospital, - - 724
Renal Affections: their Diagnosis and Pathology. By Charles Frick, M. D., - - - - - - - 724
Elementary Chemistry, Theoretical and Practical. By George Fownes, F. R. S., &c. Edited with additions, by Robert Bridges, M.D., Professor of Chemistry in the Philadelphia College of Pharmacy, &c., - - - - - - - 725

### EDITORIAL.

Western Journal of Medicine and Surgery, - - - - 725
Lectures on Diseases of the Eye, - - - - - 726
Sydenham Society of London, - - - - - 726
Medical Department of the Navy, - - - - - 726

### RECORD OF MEDICAL SCIENCE.

### PATHOLOGY AND PRACTICE OF MEDICINE.

On the Treatment of Croup by Calomel and Alum. By M. Miguel, 727
On the use of Bofareira ("Ricinus Communis" of Botanists) as a means adopted by the natives of the Cape de Verd Islands to excite Lactation. By J. O. M'William, M. D., F. R. S., R. N., Surgeon to the Honorable the Board of Customs, - - 728
Auscultatory Sign of Enlarged Liver, - - - 731

OBSTETRICS.

The Bronchocele of New-born Infants, - - - - 732

ANATOMY AND PHYSIOLOGY.

The Arrangement of the Blood-Vessels in the Mucous Membrane of the Stomach, - - - - - - - 733
On the Structure of the Muscular Substance of the Heart, - - 734
The Terminations of the Olfactory Nerve, - - - - 735

SURGERY.

On a new Method of opening Abscesses, without leaving visible Cicatrices. By M. Leriche, Physician to the Lyons Dispensary, - 735

